# Fostering international mentorship and collaborations: evaluation of the Global Bridges program for early-career researchers in health care sciences

**DOI:** 10.1186/s12909-025-07153-3

**Published:** 2025-04-26

**Authors:** Hanna Johansson, Sebastian Lindblom, Linda Timm, Paul A. Gardiner, Christina H. Opava, Ing-Mari Dohrn

**Affiliations:** 1 Division of Physiotherapy, Department of Neurobiology, Care Sciences and Society, Karolinska Institutet, Alfred Nobels Allé 23, Huddinge, 14183 Sweden; 2 Karolinska University Hospital, Theme Women’s Health and Allied Health Professionals, Stockholm, Sweden; 3 Stockholm Sjukhem Foundation, Research and Development Unit, Stockholm, Sweden; 4 Division of Family Medicine and Primary Care, Department of Neurobiology Care Sciences and Society, Karolinska Institutet, Huddinge, Sweden; 5 Division of Occupational Therapy, Department of Neurobiology Care Sciences and Society, Karolinska Institutet, Huddinge, Sweden; 6 School of Public Health, The University of Queensland, Brisbane, Australia

**Keywords:** Mentoring (MeSH), Internationalization, Learning (MeSH), Social networking (MeSH)

## Abstract

The Strategic Research Area Health Care Science was funded by the Swedish government to build strong research environments in 2008. This was assigned to Karolinska Institutet and Umeå University. A major initiative was the development of the Global Bridges program at Karolinska Institutet. The aim was to support junior researchers through mentoring and fostering international networks within health care sciences, as well as provide opportunity for them to cultivate long-lasting international collaborations essential for their careers. As participants, junior researchers were given the opportunity to invite an international scholar to Stockholm for a one-week intensive program consisting of seminars, individual mentoring sessions, and workshops. The Global Bridges program was organized six times between 2013 and 2022 with 48 junior researchers (94% women) at Karolinska Institutet and Umeå University, and 37 international scholars (68% women) from different higher education institutions in Africa, Asia, Australia, Europe, and North America. In this study, we used a mixed-method parallel design to evaluate whether the Global Bridges program had reached its intended objectives. A web-survey was sent out to all participating junior researchers and invited scholars and yielded a response rate of 71% and 83% respectively. The results indicated support for the academic development of junior researchers and that the individual components of the program were useful. Additionally, several collaborations had developed, resulting in peer-reviewed publications, conference presentations and research projects. However, more support was deemed necessary to foster long-lasting collaborations among junior researchers – invited scholar dyads.

**Supplementary Information:**

The online version contains supplementary material available at 10.1186/s12909-025-07153-3.

## Introduction

Establishing a research career is challenging. Although mentoring and collegial support are needed at all stages of an academic career [1], it is especially important for early-career researchers as they navigate their way in academia [2]. Mentoring is typically seen as the informal transmission of knowledge, social capital and psychosocial support from a person with relevant knowledge or experience (the mentor) to a person who is perceived to have less (the mentee) [3]. Successful mentoring promotes self-efficacy [4] and is an important factor when postdoctoral researchers decide whether to pursue or discontinue their academic careers [5, 6]. Although both formal (structured programs where mentor and mentee are matched by a third party) and informal (mentoring relationships that develop naturally over time) mentoring exist in academia, formal mentoring programs have been found to enhance publication productivity to a larger extent than informal mentoring [7]. Apart from providing the mentee with knowledge and support, the mentor can also serve as a gateway to academic and professional networks. Social capital and academic connections can significantly advance an individual’s career trajectory through improved opportunities for employment, publication, and conference participation [8], while also contributing value to the educational institution [9].

Internationalization is widely recognized as a vital strategy for higher education institutions as it creates opportunities for increased publications, enhanced visibility, and greater impact for researchers [10]. Therefore, internationalization is an important part of Karolinska Institutet’s (KI) Strategy 2030 [11] to develop and remain as one of the world's leading medical universities. International collaborations and networking can contribute significantly to the personal and professional development of early-career researchers, while also advancing and improving healthcare science. To achieve this, the Global Bridges program was developed with the aim of supporting junior researchers at Karolinska Institutet and Umeå University through mentoring and fostering international networks within health care sciences, as well as provide opportunity for cultivating long-lasting international collaborations essential for their careers.

In this evaluation, we describe the structure and gradual adaptations of the Global Bridges program and explore the experiences of participation from both junior researchers (mentees) and invited scholars’ (mentors) perspectives. Additionally, we map the scientific output and collaborative opportunities generated by the program. Through this evaluation we aim to explore whether the Global Bridges program is achieving its intended objectives. In doing so, we hope to guide decisions on its future direction, while also spreading learnings and examples to other institutions that may want to develop similar programs.

## Methods

### Study setting

In 2008, the Swedish Government initiated efforts in strategically selected research areas to build strong research environments of high international standards. The Strategic Research Area Health Care Science (SFO-V) was assigned to KI and Umeå University in collaboration. However, once awarded, the grant was split between the two with a smaller proportion to Umeå University with considerably fewer health care researchers than KI. Activities were developed both jointly and separately at each site. Research in health care science is essential to the advancement of knowledge in health promotion, prevention, nursing, and rehabilitation, as well as the organization and management of care. The overall vision for SFO-V is to ensure that the development and provision of healthcare services is based upon high-quality research, and the executive management team includes representatives from the local community, academia, and national health care organizations, as well as from the Stockholm and Västerbotten county councils.

### Global bridges

One of SFO-V's initiatives to advance research within health care science is the Global Bridges program, developed at KI. The program invites junior researchers in health care science to apply for the possibility to invite international scholars to KI for one week. Five to six of the applicants, with a possible one or two co-applicants, are then selected to invite an international scholar within their research area. This initiative aims to establish new contacts, share career experiences and explore potential future collaborations and mentoring opportunities. There should be no prior working relationship between the international scholar and the junior researcher’s research group. Eligible applicants are researchers in health care science at KI and Umeå University with a PhD, within 7 years after thesis defense. SFO-V provides travel and lodging expenses for the international scholars’ one-week stay in Sweden and the costs for a two-day retreat for the junior researchers and their invited scholars. Additional activities arranged for the invited scholars by the junior researchers during the week may also be funded by SFO-V.

The Global Bridges program starts with a two-day retreat. The first day consists of a joint seminar with junior researchers, invited scholars, the SFO-V management team and additional junior researchers invited by peers. During the seminar, the invited scholars share their personal career stories with a focus on career choices, work-life balance, success stories and pitfalls working as a researcher, followed by a discussion initiated by the junior researchers. On the morning of the second day, the invited scholar and the junior researcher(s) have individual mentoring meetings. In the afternoon, there is a joint wrap-up discussion and evaluation session together with SFO-V management. The following three days consist of seminars, lectures, clinical visits, workshops, social events and/or other activities agreed upon by the invited scholar and the hosting junior researcher(s). The junior researchers are expected to actively participate in planning Global Bridges program, including the joint schedule in collaboration with the SFO-V team, and organizing the individual scholars’ programs for the remaining three days. Senior and junior SFO-V peers at Umeå University are invited to participate as guests during the first day of the retreat. Junior researchers from Umeå can also be sponsored by the Umeå University SFO-V budget to participate on equal terms with their KI peers, i.e. to invite a foreign scholar for individual mentoring and further activities at Umeå University during the last three days.

**Fig. 1:**
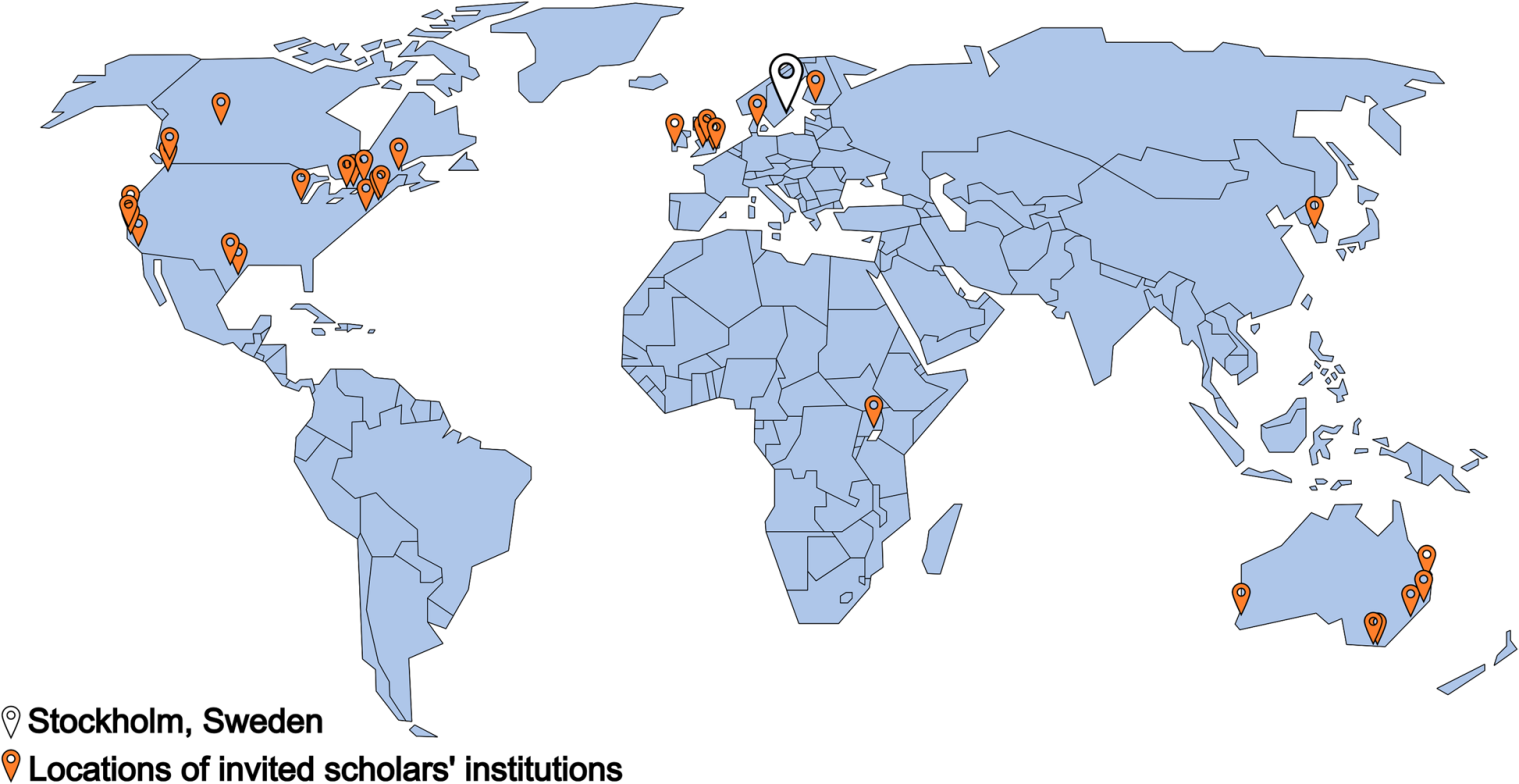
Overview of locations of invited scholar’s institutions (orange pins) and Stockholm, Sweden (white pin) where the Global Bridges program’s first week took place (the figure is modified from the image “Simple world map” by Tom-b, which is dedicated to the public domain under CC0)

Global Bridges has been arranged six times between 2013–2022 with participation of 37 international scholars (67.6% women) from Australia (*n* = 8), Canada (*n* = 9), Denmark (*n* = 1), Ireland (*n* = 1), Finland (n = 1), South Korea (*n* = 1), Uganda (*n* = 1), UK (*n* = 4) and USA (*n* = 11), and 48 junior health care science researchers (93.8% women), of which two were from Umeå University. See Fig. 1 for locations of invited scholar’s institutions on a world map.

Anecdotal evidence from conversations during Global Bridges and a short informal written evaluations of the program (2013, 2014, and 2015) found that both the junior researchers and invited scholars highly appreciated the program. Based on suggestions, a few modifications were implemented. Thus, for the last two Global Bridges (2019 and 2022) no co-applicants were included in the applications from junior researchers, however, they were still given the opportunity to invite 1–2 colleagues each to attend the first day of the program to listen to invited scholars’ presentations. Another adaptation was that the junior researchers could apply for a travel grant of up to SEK 50,000 (apx $4,900) from SFO-V to visit their invited scholar’s university within one year after participation in the Global Bridges program.

### Study design and data collection

This was a mixed-methods study with a parallel design, where qualitative and quantitative data were collected and analyzed concurrently [12]. Data were collected from junior researchers and invited scholars using online surveys. Two separate versions of the survey were developed for this study, one for invited scholars and one for junior researchers, with questions about: personal/career development, specific research activities facilitated or initiated through participation of the program, and evaluation of the program format. The questions were guided by literature about postdoctoral career progression and mentoring programs [6, 13, 14], the aims of the Global Bridges program, previous anecdotal evidence and findings from the informal evaluations of the program. The survey consisted of statements, to which the respondents were asked to agree or disagree (1 = strongly agree, 2 = agree, 3 = disagree, 4 = strongly disagree, 5 = not applicable). In addition, the respondents were asked to comment or answer open-ended questions. The survey was pilot-tested on two invited scholars and two junior researchers, and minor changes were made after feedback. The web surveys in their entirety can be found in Supplementary Information. Data were collected in August and September of 2018 (participants from 2013–2017), and again in February and March of 2024 (participants from 2019–2022), with a follow-up time ranging from one to five years after participation in the Global Bridges program. Participants were sent a link to the web-survey via email, and up to two reminder emails were sent to non-respondents.

In addition to the web-survey evaluation, a bibliometric analysis was conducted to compile the number of peer-reviewed publications co-published by participants (junior researchers and their respective invited scholar). Bibliometric analysts conducted searches on February 22, 2024, for the junior researchers in KI’s bibliometric database (which includes all publications in Web of Science and PubMed between 1995–2024). The results were screened for any co-publications with their respective invited scholar. In instances where the junior researchers did not have any publications connected to the bibliometric database, individual manual searches were conducted in the DiVA portal.

### Data analysis

Demographic data and information from the closed-ended questions of the web-survey were summarized using descriptive statistics. The open-ended questions were analyzed using thematic analysis [15]. Data from the invited scholars and junior researchers were initially analyzed separately and then merged as part of the search for patterns and meanings. The dataset was read multiple times in order to familiarize with the data. It was then coded by the second and third authors and compared in a reflexive dialogue with the first author. After that, patterns of codes with similar meanings were identified and clustered into subthemes and themes. The subthemes and themes were developed in a predominately inductive process to identify and interpret patterns of shared meanings through a reflexive dialogue between the first, second, and third authors [15]. The process was recursive to ensure that themes were meaningfully coherent, and suggestions were discussed among all authors until a consensus was reached. Three themes were formed as part of the analysis: “Opportunities for learning”, “A stepping-stone for collaboration”, and “Request for a more structured program”. Citations that best mirrored the content of each theme were selected to enhance the clarity and credibility of the analysis. These citations have been added to the manuscript without language-editing. Lastly, the results of the quantitative and qualitative analysis, as well as the bibliometric analyses were integrated during interpretation and reporting [12].

## Results

### Respondents

Thirty invited scholars responded to the survey (81.1%), and 34 responses were received from the junior researchers (70.8%). See Table 1 for an overview of participant characteristics.

**Table 1:** Characteristics of junior researcher and invited scholar respondents

	Junior researchers ***N*** = 34	Invited scholars ***N*** = 30
Female/male, n (%)	30 (88.2)/4 (11.8)	22 (73.3)/8 (26.7)
Years post-PhD, mean (SD)	2.8 (1.8)	
Appointment at time of Global Bridges, n (%)		
*Postdoctoral fellow*	25 (73.5)	
*Lecturer/senior lecturer*	2 (5.9)	4 (13.3)
*Assistant Professor*	4 (11.8)	9 (30.0)
*Associate Professor*		2 (6.7)
*Professor*		15 (50.0)
*Health care employee*	2 (5.9)	
*Other*	1 (2.9)	
Students supervised prior to Global Bridges, n (%)		
*0–11*		11 (36.7)
*12–20*		7 (23.3)
≥ *20*		12 (40.0)
Experience in mentoring junior researchers, n (%)		27 (90.0)
Prior collaboration with Karolinska Institutet, n (%)		12 (40.0)

### Impact on work-life aspects and mutual learning experiences

**Fig. 2:**
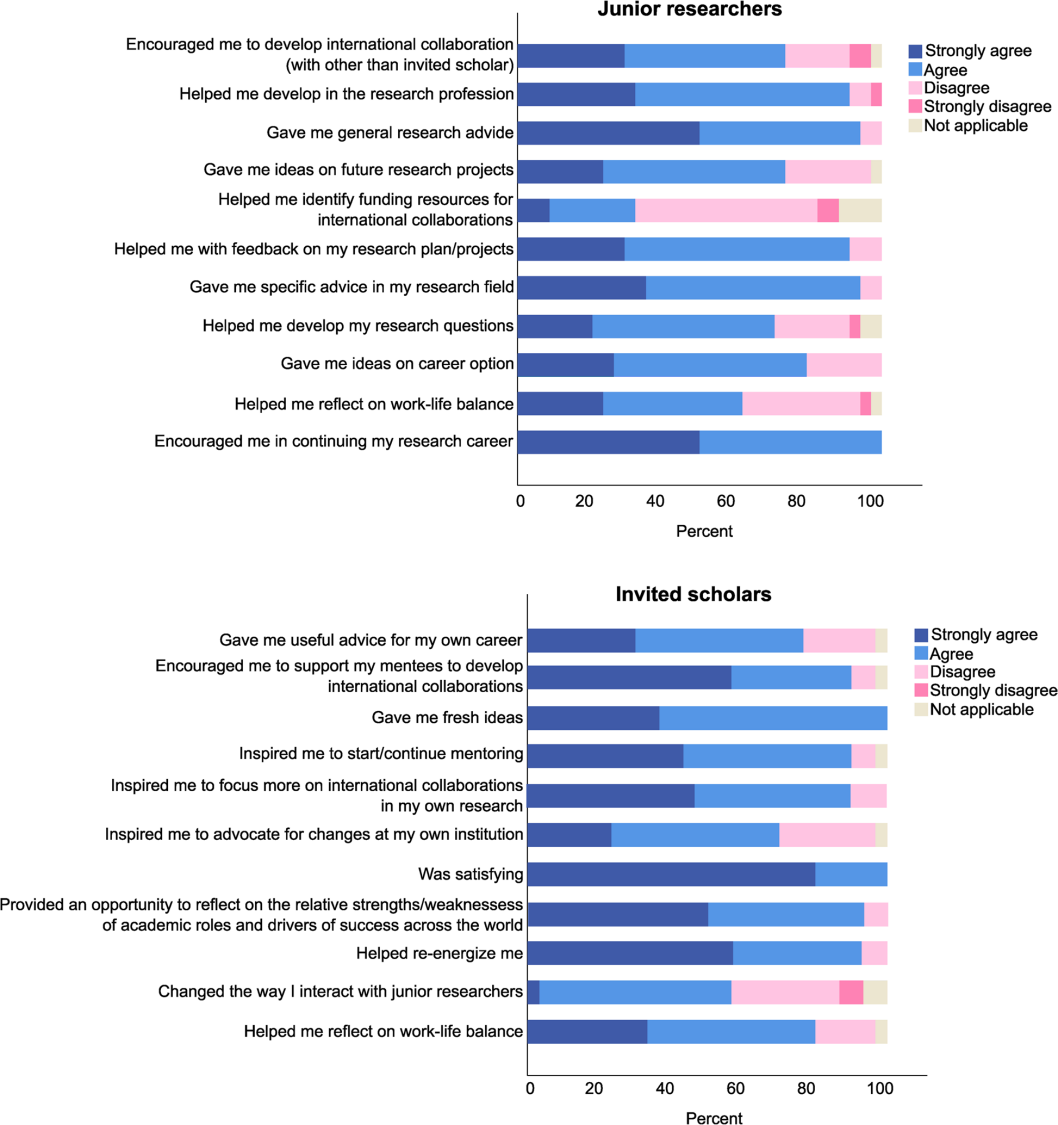
Responses from junior researchers (top) and invited scholars (bottom) to statements regarding whether Global Bridges had influenced different aspects of their work-life

In the closed-ended questions, respondents were asked to state to what extent participating in the Global Bridges program, e.g. listening to presentations, individual mentoring time, and other activities, had influenced various aspects of their work-life. The junior researchers identified that the program provided strongest support for encouragement in their research career and giving general and specific research advice with less support for provision of funding advice. All invited scholars were satisfied with the Global Bridges program, and a majority felt re-energized and inspired to start/continue mentoring. Junior researchers’ and invited scholars’ respective responses are shown in Fig. 2.

The theme “Opportunities for learning” involved how respondents described the program as an opening for both personal and professional growth, with learning occurring across all career stages. The program format, featuring one-to-one mentorship and opportunities to engage with all participating invited scholars and junior researchers, enabled learning and insights from a range of perspectives and experiences. All invited scholars appreciated sharing information, experiences, and insights, with several highlighting mutual learning as equally important.

*“I really appreciated hearing about the uniqueness of each invited scholar’s academic journey. This reaffirmed that there is no ‘one right way’ to succeed and reassured me that sometimes taking a path less travelled is not inherently riskier and can indeed be more personally fulfilling. I learned from the junior scholars and other invited scholars and participating in Global Bridges really highlighted the value of engaging in opportunities to engage with people from different places and disciplines to obtain fresh perspectives.” IS- 25.*

*“The diversity among the senior researchers was important. For me it was important to hear that there are many ways to walk the research path. The senior researchers'sharing of their research life stories was very inspiring. Being true to yourself, finding your passion and path in research was crucial but to be successful in research you also have to be in a supportive environment with opportunities and a system that supports you.” JR- 13.*

Junior researchers reported multiple learning opportunities throughout the program. The one-to-one mentorship provided advice and offered guidance on career development and strategies for building academic resilience. It further facilitated reflections on goals and future directions in dialogue between mentor and mentee.

*“It was great to have one-to-one time to reflect and discuss career development. I was challenged to reflect on my possibilities and obstacles.” JR- 3*

Even though the opportunity to observe the mentor’s approach to work and private life provided learnings in what to aspire to, it also gave insights and an understanding of paths to avoid in relation to career and personal development.

*“My own reflection when I saw how my invited senior researcher worked (working all days except for Christmas Day, available and responding to email every waking hour) that I want to find another balance between working and private life. I don’t aim for her level, although I was very inspired in other ways.” JR- 2.*

### International collaborations: achievements and challenges

Twelve junior scholars (35.3%) reported having collaborated on a grant application with their invited scholar after participating in Global Bridges. At the time of the survey, two grant applications had been approved/funded. Seven junior scholars (20.6%) had worked with their invited scholar on reviewing a manuscript, and 15 (44.1%) had collaborated with their scholar on writing a manuscript (7 published and 8 in stage of preparation at time of survey). Sixteen junior researchers (47.1%) reported that they had collaborated with their invited scholar on a new study. Four of these studies had started, and one was finished. Most of these collaborative studies (nine) took place at KI, two at the invited scholar’s university, and one was multi-site. Eight junior researchers (23.5%) stated having collaborated with their scholar on a conference abstract, and four (11.8%) that they had been in contact with their respective scholar to be part of an international expert committee or panel. At the time of the survey, 16 (47%) of the junior researchers had either planned (*n* = 3) or completed (*n* = 13) a visit at the invited scholars’ university. Searches in the KI bibliometric database and manual searches in DiVA yielded a total of 1260 publications. Of these, 17 were co-publications between the Global Bridges program participant dyads.

The theme “A stepping-stone for collaboration” involved respondents describing the Global Bridges program as a valuable opportunity and a springboard for future collaboration. The extent of these collaborations varied: while some evolved into active and extensive partnerships, others concluded without further contact or collaboration. For some respondents, the program facilitated different forms of fruitful collaboration, including research projects, publications, grant applications, educational initiatives, conferences, research visits, and continued mentorship.

*“Global bridges started a close collaboration with my invited scholar and resulted in a 3-month postdoc visit at his institution. During this visit I had the opportunity to start networking, and collaborations with other international researchers introduced to me by my invited scholar. These contacts have been very helpful in my own specific research project, and I am sure they also will be helpful in my future research career.” JR- 15.*

Conversely, some respondents faced challenges in maintaining contact and continuing collaboration after the Global Bridges program ended, with some experiencing no further contact at all. Reasons for this varied, including time and resource constraints, institutional barriers, lack of interest, differences in research interests, and significant life events such as parental leave, retirement, or transitioning to a non-academic role.

*“The potential for collaboration with my invited scholar was very good, but unfortunately"mechanisms"… and demonstrations of power at our respective universities blocked further collaboration. The invited scholar said"Yes"to the question to be my mentor, which I really would have appreciated, but in practice she was not interested at all.” JR- 11”*

*The lack of contact sometimes led to feelings of disappointment*

*“I actually have been mildly surprised and disappointed that the junior researcher did not reach out to me for further feedback, and never even notified me when she published manuscripts relevant to her project. I thought I had been very generous with time and expertise.” IS- 16.*

Some respondents described participation in the Global Bridges program as an opportunity to extend their network and collaborate beyond the established mentor–mentee contact.

*“The major research activities that have been facilitated through participation in Global Bridges have centered on specific and important research collaborations with some of the Swedish faculty that attended Global Bridges, some of whom had formerly been at Karolinska. This has been fantastic and a wonderful benefit of the Global Bridges program. Through those researchers I have been able to extend my Swedish research collaborations with more junior researchers in their group as well.” IS- 27.*

### Respondent perspectives on program activities and structure

**Fig. 3:**
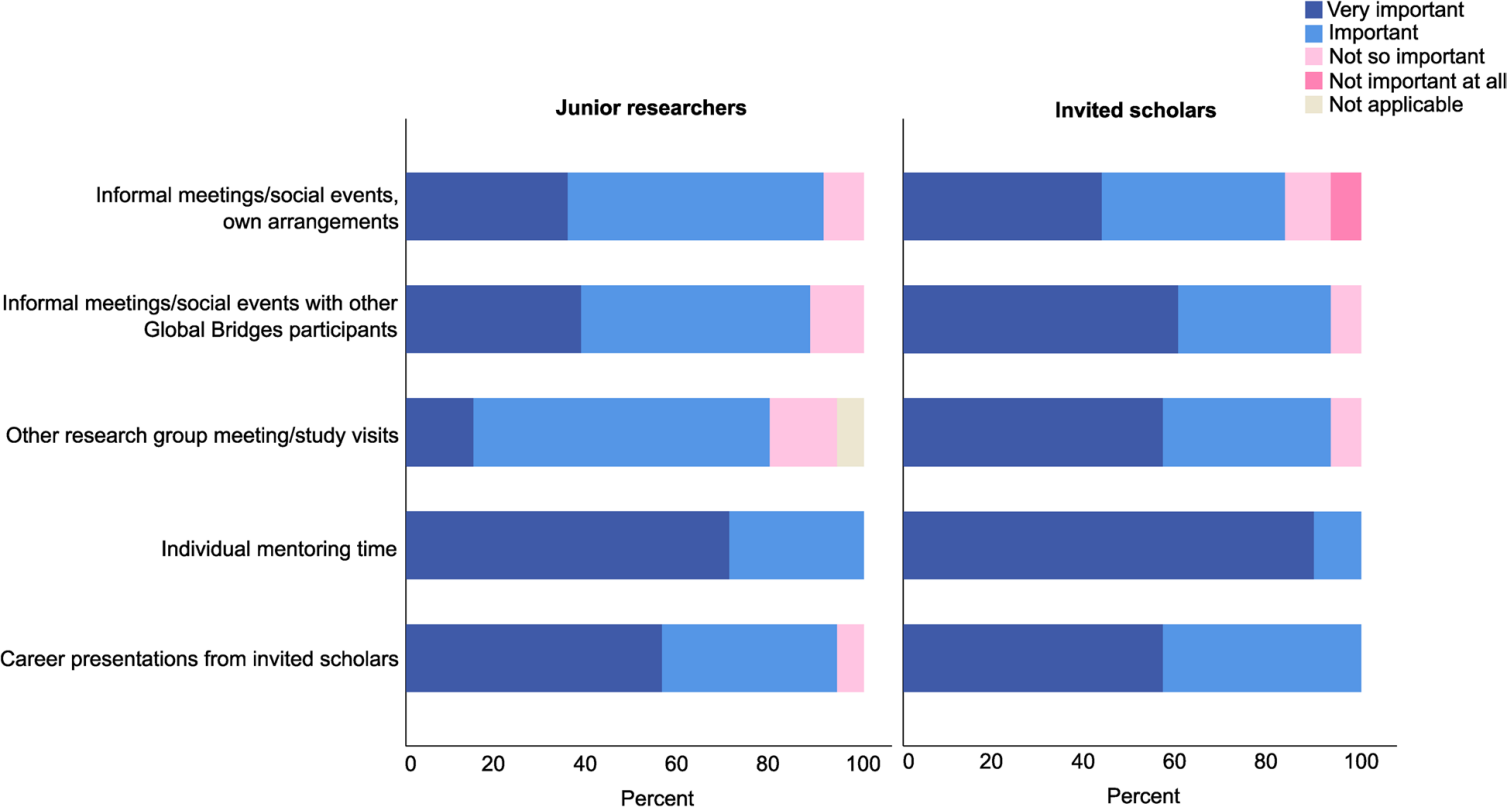
Responses from junior researchers (left) and invited scholars (right) regarding how important they believed that the various activities of the Global Bridges program had been in order to reach the intended aims

Respondents were further asked how important they believed that the various activities during the week had been in order to reach the aims of the Global Bridges program (i.e., facilitate the building of networks and future collaborations between junior researchers and international scholars). Both junior researchers and invited scholars identified individual mentoring sessions as the most valuable activity, with all participants rating them as important or very important. Informal meetings, social events, and study visits were viewed as somewhat less important by both groups. Figure 3 illustrates the ratings given by junior researchers and invited scholars.

When asked whether the Global Bridges program had met their expectations, 50 (78.1%) of respondents answered *yes*, and 14 (21.9%) answered *yes to some* extent (no respondents responded *no*). All respondents stated that they would recommend other junior researchers/research scholars to take part in the program.

Even though the respondents greatly valued the program, more structure was suggested to clarify its aims and expectations and to better support continued collaboration. As evident in the theme “Request for a more structured program”, some junior researchers expressed a need for greater clarity regarding the program's objectives and expectations to facilitate dialogue on the roles of invited scholars and the intended outcomes of the mentorship.

*“It is a great opportunity but it could be a bit sharper in the aim of the program. For example, what were the expectations on further collaboration versus career guidance during the week.” JR- 3*

Both the invited scholars and junior researchers reported a need to formalize a plan and structure for follow-up and continued collaboration.

*“I think there could be some type of formalized follow up after the visit. There are probably some on-line activities that could be facilitated to help build and maintain the relationship. Just need to get people started.” IS- 1*

*“It was a wonderful program and well facilitated. It may be good to provide the mentees with a bit more structure to following up with their mentor once the intensive week was over.” IS- 5*

## Discussion

This evaluation aimed to explore whether the Global Bridges program achieved its intended objectives, i.e., supporting the development of junior researchers at KI through mentoring, fostering international networks within health care sciences, and cultivating long-lasting international collaborations essential for their careers. Our results indicated that the program was highly valued by both junior researchers and invited scholars, with individual mentoring sessions identified as the most important component. This could possibly be explained by the fact that individual mentoring helps promote self-efficacy [4] and acts as a catalyst for career and leadership enhancement [16], factors important when trying to establish a career in academia. Previous research has also shown that having a mentor from a different institution can help remove power differentials and foster mutual learning [6]. In addition, several of the invited scholars highlighted the mutual learning opportunity of engaging as a mentor, together with the possibility for self-reflection and satisfaction of helping others, benefits previously described in the literature [17].

A mentor relationship evolves through four phases; initiation, cultivation, separation and redefinition [18]. As the junior researchers and the invited scholars were getting to know each other during the week in Stockholm, this was part of the initiation phase. The organizers deliberately did not decide on the format or content to be discussed during the individual mentoring time, and some participants expressed that they would have wanted guidelines for these sessions. Establishing clear mentorship goals has been described as an essential part of a successful mentorship [19], hence is something to consider in future development of the program. Aligned with setting goals is also understanding the expectations that the mentor and the mentee have on the mentoring relationship, and whether these expectations are compatible. Previous research has for example shown that mentees in academia typically value social support behavior from their mentor, whereas mentors instead value career-related behaviors in their mentee [20]. By formalizing discussions on goals and expectations, both junior researchers and invited scholars would also be given the opportunity to rectify incompatible expectations.

A significant portion of the first day of the program was dedicated to presentations by the invited scholars. They shared their personal career stories with an emphasis on career choices rather than their actual research outputs. This segment was highly appreciated by both junior researchers and the invited scholars themselves, as it clearly demonstrated that there is no single path to achieving an academic career, while also acknowledging the challenges of work-life balance and family responsibilities. With increased turnover rates and work-related mental health concerns among early-career researchers around the world [21], finding ways to support and retain talented faculty is important for universities. Although the reasons for choosing to stay in academia are highly individual and diverse, intellectual freedom and maintaining a good work-life balance seem to be important drivers [22].

Internationalization is regarded as important for advancing both research and education at universities. Junior researchers are often encouraged to spend time abroad to strengthen their CVs and enhance their career prospects upon returning. However, this may not always be feasible due to personal circumstances, highlighting the need for additional ways to establish international collaborations and networks in health care sciences. Over the ten-year period, the Global Bridges program has had a broad international reach, with invited scholars from five different continents. This has enabled junior researchers to initiate a new international collaboration independent from their research group at KI, something which is of high importance for personal academic growth. Notably, our results suggested that these opportunities for collaborative research, publishing, and expanding international networks benefitted not only the junior researchers but also the invited scholars.

Although not all mentee-mentor dyads resulted in sustained contact or future research collaborations, our evaluation indicated that the program could foster long-lasting connections and lead to research outcomes, such as publications. Our bibliometric analyses identified 15 publications co-authored by a Global Bridges dyad, with several involving the same dyad. However, because our searches did not extend beyond these dyads, it is possible that other collaborations generated by the Global Bridges program were missed. For example, manuscripts co-authored by the junior researcher with other researchers introduced by the invited scholar would not have been captured in our analysis. Given that some participants received the web-survey less than two years after participation in the program, they may not have been able to publish within that timeframe. Although funding plays an important role in initiating new collaborations, time is required for these collaborations to become effective and productive [23]. Further, although networking has been shown to increase the number of citations, it does not necessarily have any bearing on the quality or quantity of scientific publications [24]. It is also important to remember that although publications and number of citations are tangible outputs of research activity, they are not the only indicators (others include, but are not limited to, grant applications, conference proceedings, posters, patents, digital and visual media, etc.).

As answers to the open-ended questions showed, both junior researchers and invited scholars expressed a need for the Global Bridges program to be more structured. A recurring suggestion was that the aims be made more explicit both to junior applicants and to their invited scholars already at the invitation stage. As suggested by Treasure et al. [25], a clear vision and well-defined scope and outcomes is the foundation needed to inform all design aspects of a mentorship program. This could ensure that the expectations of future participants are aligned with the intentions of the program, and help invited scholars to make a more informed decision as to whether they have the capacity and will to mentor and/or collaborate with the junior researcher. It was further suggested that the junior researcher be given advice about factors important to consider when inviting a suitable researcher. Given a focus on long-lasting collaboration for example, researchers close to retirement may not be the best choice for invited scholars. One of the prerequisites for a junior researcher’s acceptance into the program was that they should not have had any prior collaborations with the invited scholar, and if any contact had occurred, it should have been only brief prior to the invitation. This concept has remained unchanged in the program's development, as it was one of the main objectives to support new collaborations and networks beyond the contacts already established within the junior researchers'research groups. Given how some dyads struggled to maintain contact after the Global Bridges program ended, it does however highlight the importance of supporting the participants in forming personal connections between the mentor and the mentee during the initiation phase in Stockholm. This evaluation also revealed that participants requested a more formal follow-up after the week in Stockholm. Perhaps the Global Bridges program needs to reposition itself as an ongoing program supporting the dyads throughout the different mentoring phases [18] and not just a one-week intensive program focused on initiation. A more formalized mentoring structure could be adopted once the invited scholars leave Sweden to ensure ongoing contact [26]. Indeed, one such adaptation which has been in place since 2022 is the possibility for junior researchers to apply for a travel grant to visit their respective invited scholar’s institution.

Another aspect of note is that a majority of the participants, both junior researchers and invited scholars, were women. Ninety-four percent of the participating junior researchers were women, and this was more than 20 percent higher than the mean percentage of women post-doctoral researchers at the concerned department at KI at the six different Global Bridges time-points. Gender disparities and gender bias in academia is widely recognized, and affects all levels including scientific productivity, funding opportunities and academic promotion [27]. Despite women in academia seeing mentoring as more important than men, they are less likely to have a mentor [28]. Women also tend to have access to strategic networks to a lower degree than men [8]. Although the Global Bridges program was not intended to give specific opportunities to women, it does seem to have served an important purpose given the number of women applicants and women invitees.

### Limitations

Some methodological limitations should be acknowledged. The survey, which relied on retrospective self-report data, was conducted up to five years after participation in the Global Bridges program. Indeed, some respondents expressed difficulties in recalling their experiences in relation to certain items included in the survey. Over time, external factors other than the Global Bridges program have most likely also influenced the career trajectories of both junior researchers and invited scholars. However, it is necessary to allow time for further development of possible collaborations to take place, such as grant applications, visits and publications. One way to overcome this limitation might be to conduct a first survey on short-term benefits using some questions from this survey within six months after the visit to Sweden. Another survey could then be sent out three years later to capture specific research outcomes. Further, the application process into Global Bridges increased the risk of self-selection bias by attracting participants who sought and were positive toward mentorship. Future research intended to evaluate mentorship programs could therefore benefit from using a control group design. Lastly, as the program has evolved over time, the participants have not all experienced the same version of the program. The responsiveness of the organizers (i.e., SFO-V) to update and improve the program based on feedback from participants should however also be considered a strength.

## Conclusion

The Global Bridges program has positively impacted junior researchers and invited scholars by providing an opportunity for mentoring and fostering international networks and collaboration in healthcare sciences. While the program was highly appreciated among participants and has led to successful collaborations and publications, more support is needed for long-lasting partnerships between junior researchers and invited scholars. More explicit program aims, and a more structured approach could improve the decision-making process for invited scholars and enhance collaboration sustainability. Repositioning the Global Bridges program as an ongoing initiative rather than a one-week intensive program could better support junior researchers and foster enduring international collaborations.

## Supplementary Information

Supplementary Material 1.

## Data Availability

The datasets analysed during the current study are available from the corresponding author on reasonable request.
