# Supplementary material for: Fostering international mentorship and collaborations: evaluation of the Global Bridges program for early-career researchers in health care sciences
Source: BMC Med Educ. 2025 Apr 26;25:616. doi: 10.1186/s12909-025-07153-3 (PMC12032714; doi:10.1186/s12909-025-07153-3)
Supplement: Supplementary file 1 — Supplementary Material 1. [file 12909_2025_7153_MOESM1_ESM.pdf]

Evaluation of Global Bridges

Web survey

Version: Invited scholars

Questions regarding personal/career development

Participating in the Global Bridges program, e.g. preparing my presentation, listening to the other invited scholars' presentations, having individual mentoring time with junior researchers, and other activities during the week (including research site and hospital visits)…

	strongly agree	agree	disagree	strongly disagree	Not applicable
Helped me reflect on work-life balance	☐	☐	☐	☐	☐
Changed the way I interact with junior researchers	☐	☐	☐	☐	☐
Helped re-energize me	☐	☐	☐	☐	☐
Gave me useful advice for my own career	☐	☐	☐	☐	☐
Was satisfying	☐	☐	☐	☐	☐
Inspired me to advocate for changes at my own institution	☐	☐	☐	☐	☐
Gave me fresh ideas	☐	☐	☐	☐	☐
Inspired me to start/continue mentoring	☐	☐	☐	☐	☐
Inspired me to focus more on international collaborations in my own research	☐	☐	☐	☐	☐
Encouraged me to support my mentees to develop international collaborations	☐	☐	☐	☐	☐
Provided an opportunity to reflect on the relative strengths/weaknesses of academic roles and drivers of success across the world	☐	☐	☐	☐	☐

What advice or piece of information from the other invited scholars was most useful?

Questions regarding specific research activities

Since the Global Bridges, have you had contact with the junior researcher(s) to collaborate on a grant application?

☐ Yes

☐ No

Comment

What is the status of the grant application?

☐ Submitted

☐ Received

☐ Don't know

☐ If other, please specify

Since the Global Bridges, have you had contact with the junior researcher(s) to review a manuscript?

☐ Yes

☐ No

Comment

Since the Global Bridges, have you had contact with the junior researcher(s) to collaborate on a manuscript?

☐ Yes

☐ No

Comment

What is the status of the manuscript?

- [ ] Submitted
- [ ] Published
- [ ] Don't know
- [ ] If other, please specify

Since the Global Bridges, have you discussed with the junior researcher(s) to collaborate on a new study?

- [ ] Yes
- [ ] No

Comment

What is the status of the discussion concerning the new study?

- [ ] The study is planned
- [ ] The study has started
- [ ] The study has finished
- [ ] Don't know

Comment

Where is this collaboration mainly occurring?

- [ ] Karolinska Institutet
- [ ] Invited scholar’s university
- [ ] Internationally/Multi-center
- [ ] If other, please specify

Comment

Since the Global Bridges, have you had contact with the junior researcher(s) to collaborate on a conference abstract/seminar?

☐ Yes

☐ No

Comment

Has this conference abstract been:

☐ Submitted

☐ Accepted

☐ Done

☐ Don't know

Comment

Since the Global Bridges, have you had contact with the junior researcher(s) to to visit her/his research group?

☐ Yes

☐ No

Comment

What is the status of the visit?

☐ The visit is planned

☐ The visit has taken place

☐ Don't know

Comment

Since the Global Bridges, have you had contact with the junior researcher(s) to be a part of international expert panels or committees?

☐ Yes

☐ No

Comment

Please list any other research activities that have been facilitated through participation in Global Bridges at:

Karolinska Institutet

Other institutions in Sweden

Internationally

Additional comments on this area

Questions regarding the evaluation of the program

The aims of Global Bridges are to facilitate the building of networks and future collaboration between junior researchers and international scholars. To reach these aims, in your opinion how important were:

	Very important	Important	Not so important	Not important at all	Not applicable
Career presentations from invited scholars	☐	☐	☐	☐	☐
Individual mentoring time	☐	☐	☐	☐	☐
Other research group meetings/study visits	☐	☐	☐	☐	☐
Informal meetings/social events with other Global Bridges participants	☐	☐	☐	☐	☐
Informal meetings/social events, own arrangements	☐	☐	☐	☐	☐

Comment

Global Bridges met my expectations:

☐ Yes

☐ Yes, to some extent

☐ No

☐ Don't know

Would you recommend other scholars researchers to participate in Global Bridges?

☐ Yes

☐ No

☐ Don't know

Do you have any suggestions on how to improve Global Bridges?

Questions regarding background characteristics

1. What is your gender?

☐ Female

☐ Male

What year were you born in?

What was your work position at the time of Global Bridges?

☐ Postdoctoral fellow

☐ Assistant professor

☐ Lecturer/Senior lecturer

☐ University administration

☐ Health care employee

☐ If other, please specify

Comment

Prior to Global Bridges, what was the number of students supervised?

Comment

Prior to Global Bridges, did you have experience in mentoring junior researchers regarding career?

☐ Yes

☐ No

Comment

Prior to Global Bridges, have you collaborated with anyone from KI?

☐ Yes

☐ No

Comment

Do you have any other comments or suggestions regarding the evaluation survey?

Thank you for your feedback!

Evaluation of Global Bridges

Web survey

Version: Junior researchers

Questions regarding personal/career development

Participating in the Global Bridges program, e.g. listening to presentations, having individual mentoring time with invited scholars, and other activities during the week (including research site and hospital visits)…

	strongly agree	agree	disagree	strongly disagree	Not applicable
Encouraged me in continuing my research career	☐	☐	☐	☐	☐
Helped me reflect on work-life balance	☐	☐	☐	☐	☐
Gave me ideas on career options	☐	☐	☐	☐	☐
Helped me develop my research questions	☐	☐	☐	☐	☐
Gave me specific advice in my research field	☐	☐	☐	☐	☐
Helped me with feedback on my own research plans/projects	☐	☐	☐	☐	☐
Helped me identify funding resources for international collaborations	☐	☐	☐	☐	☐
Gave me new ideas on future research projects	☐	☐	☐	☐	☐
Gave me general research advice	☐	☐	☐	☐	☐
Helped me develop in the research profession	☐	☐	☐	☐	☐
Encouraged me to develop international collaborations (with other than invited scholars)	☐	☐	☐	☐	☐

Comment

What was the best advice or piece of information you heard or received during Global Bridges?

Questions regarding specific research activities

Since the Global Bridges, have you had contact with your invited scholar to collaborate on a grant application?

- [ ] Yes
- [ ] No

Comment

What is the status of the grant application?

- [ ] Submitted
- [ ] Received
- [ ] Don't know
- [ ] If other, please specify

Since the Global Bridges, have you had contact with your invited scholar to review a manuscript?

- [ ] Yes
- [ ] No

Comment

Since the Global Bridges, have you had contact with your invited scholar to collaborate on a manuscript?

☐ Yes

☐ No

Comment

What is the status of the manuscript?

☐ Submitted

☐ Published

☐ Don't know

☐ If other, please specify

Comment

Since the Global Bridges, have you discussed with your invited scholar to collaborate on a new study?

☐ Yes

☐ No

Comment

What is the status of the discussion concerning the new study?

☐ The study is planned

☐ The study has started

☐ The study has finished

☐ Don't know

Comment

Where is this study collaboration mainly occurring?

☐ Karolinska Institutet

☐ Invited scholar's university

☐ Internationally/Multi-center

☐ Other

Comment

Since the Global Bridges, have you had contact with your invited scholars to collaborate on conference abstracts/seminars?

☐ Yes

☐ No

Comment

Has the conference abstract been:

☐ Submitted

☐ Accepted

☐ Done

☐ Don't know

Comment

Since the Global Bridges, have you had contact with your invited scholar to visit her/his research group?

☐ Yes

☐ No

Comment

What is the status of the visit?

☐ The visit is planned

☐ The visit has taken place

☐ Don't know

Comment

Since the Global Bridges, have you had contact with your invited scholar to be a part of international expert panels or committees?

☐ Yes

☐ No

Comment

Please list any other research activities that have been facilitated through participation in Global Bridges at:

Karolinska Institutet

Other institutions in Sweden

Internationally

Additional comments on this area

Questions regarding the evaluation of the program

The aims of Global Bridges are to facilitate the building of networks and future collaboration between junior researchers and international scholars. To reach these aims, in your opinion how important were:

	Very important	Important	Not so important	Not important at all	Not applicable
Career presentations from invited scholars	☐	☐	☐	☐	☐
Individual mentoring time	☐	☐	☐	☐	☐
Other research group meetings/study visits	☐	☐	☐	☐	☐
Informal meetings/social events with other Global Bridges participants	☐	☐	☐	☐	☐
Informal meetings/social events, own arrangements	☐	☐	☐	☐	☐

Comment

Global Bridges met my expectations:

☐ Yes

☐ Yes, to some extent

☐ No

☐ Don't know

Would you recommend other junior researchers or research scholars to participate in Global Bridges?

☐ Yes

☐ No

☐ Don't know

Do you have any suggestions on how to improve Global Bridges?

Questions regarding background characteristics

What is your gender?

☐ Female

☐ Male

☐ Other

What year were you born in?

What is your professional/university degree?

What year did you receive your PhD?

What year did you participate in Global Bridges?

What was your work position at the time of Global Bridges?

☐ Postdoctoral fellow

☐ Assistant professor

☐ Lecturer/Senior lecturer

☐ University administration

☐ Health care employee

☐ If other, please specify

Comment

What is your current work position?

☐ Postdoctoral fellow

☐ Assistant professor

☐ Lecturer/Senior lecturer

☐ University administration

☐ Health care employee

☐ If other, please specify

Comment

Do you have any other comments or suggestions regarding the evaluation survey?

Thank you for your feedback!
